# Targeting Regulatory T Cells by Addressing Tumor Necrosis Factor and Its Receptors in Allogeneic Hematopoietic Cell Transplantation and Cancer

**DOI:** 10.3389/fimmu.2019.02040

**Published:** 2019-08-28

**Authors:** Harald Wajant, Andreas Beilhack

**Affiliations:** ^1^Division of Molecular Internal Medicine, Department of Internal Medicine II, University Hospital Würzburg, Würzburg, Germany; ^2^Department of Internal Medicine II, University Hospital Würzburg, Würzburg, Germany; ^3^Center for Interdisciplinary Clinical Research, University of Würzburg, Würzburg, Germany; ^4^Else-Kröner-Forschungskolleg Würzburg, Würzburg University Hospital, Würzburg University, Würzburg, Germany

**Keywords:** GVHD, graft vs. host disease, cancer, Tregs (regulatory T cells), TNFR family costimulatory receptors, TNFR2 agonists, TNFR2 antagonism

## Abstract

An intricate network of molecular and cellular actors orchestrates the delicate balance between effector immune responses and immune tolerance. The pleiotropic cytokine tumor necrosis factor-alpha (TNF) proves as a pivotal protagonist promoting but also suppressing immune responses. These opposite actions are accomplished through specialist cell types responding to TNF via TNF receptors TNFR1 and TNFR2. Recent findings highlight the importance of TNFR2 as a key regulator of activated natural FoxP3^+^ regulatory T cells (Tregs) in inflammatory conditions, such as acute graft-vs.-host disease (GvHD) and the tumor microenvironment. Here we review recent advances in our understanding of TNFR2 signaling in T cells and discuss how these can reconcile seemingly conflicting observations when manipulating TNF and TNFRs. As TNFR2 emerges as a new and attractive target we furthermore pinpoint strategies and potential pitfalls for therapeutic targeting of TNFR2 for cancer treatment and immune tolerance after allogeneic hematopoietic cell transplantation.

## Introduction

Tumor necrosis factor-alpha (TNF) regulates innate as well as adaptive immune processes and controls tissue homeostasis in various ways. TNF reached prominence as a prototypic proinflammatory cytokine, however, more recently, the TNF-TNF receptor system gained attention for its immunomodulatory and even anti-inflammatory functions. Here, we review important activities of TNF and its receptors crucial for T cell and Treg function under pathologic conditions such as acute graft-vs.-host disease (GvHD). The implications of the molecular basis of TNF receptor signaling are then discussed for the rational development of therapeutic TNFR-receptor-targeting reagents for clinical applications.

## General Aspects of TNFR1 and TNFR2 Signaling

**Figure 1:**
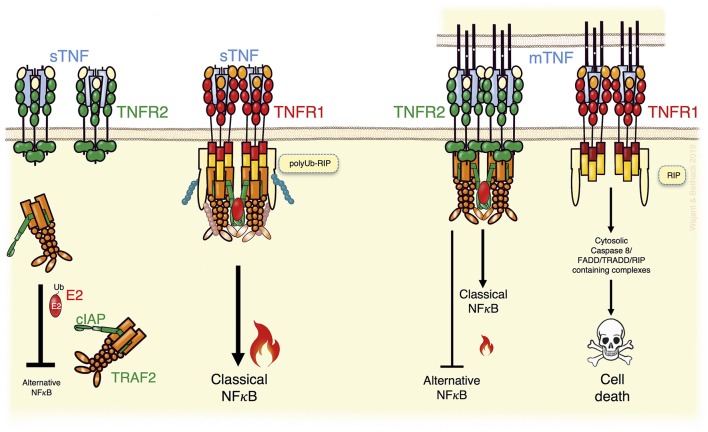
TNFR2 can modulate TNFR1 signaling. TNFR1, activated by soluble TNF (sTNF) or membrane TNF (memTNF), recruits TRAF2 adapter protein trimers enabling transactivation of the TRAF2-associated E3 ligases cIAP1 and cIAP2 and activation of the classical NFκB pathway but also other proinflammatory signaling pathways not indicated here (**Left**). In addition, in TNFR1 signaling the TRAF2-cIAP1/2 complexes inhibit triggering of cell death by K63 ubiquitination of RIP (**Left**). TNFR2 activation by memTNF recruits TRAF2-cIAP1/2 complexes, too, and triggers classical NFκB signaling (**Right**). Due to the higher expression levels of TNFR2 and its ability to trigger TRAF2 degradation, however, TNFR2 activation can result in a substantial depletion of cytosolic TRAF2-cIAP1/2 complexes (**Right**). This entails enhanced alternative NFκB signaling and sensitizes for TNFR1-induced death signaling. For details see text.

TNF is a single spanning type II transmembrane protein and the name giving member of the TNF superfamily (TNFSF) (1). TNF and the other ligands of the TNFSF share a conserved C-terminal homology domain, the TNF homology domain (THD), which mediates self-assembly into trimeric molecules and receptor binding. A short stalk region connects the THD of TNF with the transmembrane and the cytoplasmic domain. Membrane TNF (memTNF) can be cleaved in its stalk region by the metalloprotease TNFα converting enzyme (TACE, ADAM17) resulting in the release of trimeric soluble TNF (sTNF) (2). Both forms of TNF are able to bind to two receptors, TNF receptor-1 (TNFR1) and TNFR2, which belong to the TNF receptor superfamily (TNFRSF). A trimeric TNF molecule interacts with three molecules of either TNFR1 or TNFR2 (3). Importantly, memTNF activates both TNF receptors, while only TNFR1 responds strongly to sTNF (Figure 1) (4). Triggering of TNFR2-associated signaling pathways requires secondary clustering of initially formed trimeric TNF-TNFR2 complexes. This occurs spontaneously for memTNF-induced TNF-TNFR2 complexes but not sTNF-liganded TNFR2 complexes (Figure 1) (5). Lymphotoxin-alpha (LTα), another soluble ligand trimer of the TNFSF, also interacts with the two TNF receptors triggering similar effects as sTNF.

TNFR1 and TNFR2 can be assigned to two different subgroups of the TNFRSF. TNFR1 belongs to the TNFRSF death receptor subgroup. Death receptors are characterized by a cytoplasmic protein-protein interaction domain called death domain (DD), which enables these receptors to trigger cytotoxic signaling (1). TNFR2, on the other side, is a representative of the TNF receptor associated factor (TRAF)-interacting receptor subgroup of the TNFRSF. Therefore, TNFR2 lacks a death domain and instead directly interacts with TRAF family members, which form homo- or heterotrimers (6). Although TNFR1 can trigger apoptotic and necroptotic signaling via its DD, these cytotoxic activities are not prevalent. They are typically inhibited by Fas-associated death domain (FADD)-like IL-1β-converting enzyme-inhibitory proteins (FLIPs) and/or complexes of a TRAF2 trimer and a single cellular inhibitor of apoptosis-1 (cIAP1) or cIAP2 E3 ligase molecule (7, 8). Accordingly, TNFR1 stimulation results primarily in the engagement of cell death-independent proinflammatory pathways activating NFκB transcription factors and MAP kinases. Notably, TRAF2 and the cIAPs not only antagonize cytotoxic TNFR1 signaling but also contribute to TNFR1-induced proinflammatory signaling (9). The TRAF2-cIAP1 and TRAF2-cIAP2 complexes are indirectly recruited to trimeric TNF-TNFR1 complexes by the DD-containing adapter protein TNF receptor associated death domain (TRADD). In context of the TNFR1 signaling complex, the TRAF2-cIAP1/2 complexes K63-ubiquitinate the DD-containing serine/threonine kinase receptor interacting protein (RIP), which is recruited via its DD to the DD of TNFR1 independently from TRADD. K63-ubiquitinated TNFR1-bound RIP creates docking sites for the linear ubiquitin chain assembly complex (LUBAC) and for various K63 or linear ubiquitin binding-domain containing signaling intermediates, such as the NFκB essential modulator (NEMO) subunit of the inhibitor of kappaB kinase (IKK) complex and the TGF-beta activated kinase-1 (TAK1) binding protein-2 (TAB2) subunit of the IKK-activating TAB2-TAK1 complex (7, 9). Thus, K63-ubiquitination of RIP strongly enhances the TRAF2-dependent ability of the TNFR1-TRADD-TRAF2-cIAP1/2 core complex to recruit the IKK complex and the TAB2-TAK1 complex. This leads to IKK-mediated phosphorylation of the inhibitor of kappaB-alpha (IκBα), its proteasomal degradation and the subsequent nuclear translocation of previously IκBα-sequestered dimers of the NFκB transcription factor family. Furthermore, K63-ubiquitination of TNFR1-associated RIP antagonizes the ability of the latter to trigger apoptosis and necroptosis. The initiation of these cell death responses is based on the release of RIP from the TNFR1 signaling complex and its subsequent interactions with caspase-8 and/or RIP3 in cytosolic complexes (7, 8). TNFR1-induced RIP-mediated caspase-8 activation results in apoptosis, while the interplay of RIP with RIP3 may stimulate necroptosis. Since caspase-8 actively suppresses necroptotic signaling, e.g., by cleavage of RIP and RIP3, TNF-induced necroptotic signaling typically becomes only relevant in cells with a compromised ability to activate caspase-8 (7, 8).

Interestingly, TNFR2 recruits very efficiently TRAF2-cIAP1/2 complexes (Figure 1). Indeed, TRAF2 and the cIAPs were originally identified as TNFR2 signaling components (10, 11) and are relevant for TNFR2-induced classical NFκB signaling, too. Since TNFR2 is typically much higher expressed as TNFR1, recruitment of TRAF2-cIAP1/2 complexes to TNFR2, but not to TNFR1, reduces the freely available cytoplasmic pool of these molecules (Figure 1) (12). Moreover, TNFR2-mediated depletion of cytosolic TRAF2-cIAP1/2 complexes can be enhanced by TNFR2-stimulated TRAF2 proteolysis. Since the cytosolic TRAF2-cIAP1/2 complexes contribute to constitutive MAP3K NFκB inducing kinase (NIK) degradation, sequestration, and degradation of TRAF2-cIAP1/2 complexes by TNFR2 result in NIK accumulation. NIK has a high basal activity and, therefore, NIK accumulation already triggers phosphorylation of NIK substrates. NIK's best investigated substrate is the p100 precursor protein of the p52 NFκB transcription factor subunit. NIK-mediated p100 phosphorylation promotes limited proteolysis to p52. This triggers the conversion of cytoplasmic p100-containing NFκB dimers to p52-containing dimers, which can translocate into the nucleus. In contrast to TNFR1, TNFR2 can therefore not only stimulate nuclear translocation of NFκB dimers by the IKK complex-dependent classical pathway but also by an alternative pathway based on IKK complex-independent p100 processing (5). TNFR2-mediated sequestration/degradation of TRAF2 complexes not only affects the inhibitory effect of the TRAF2-cIAP1/2 complexes on the alternative NFκB pathway but also limits their availability for TNFR1 (Figure 1). Consequently, TNFR2 activation can attenuate TNFR1-induced classical NFκB signaling and sensitize cells for TNFR1-induced cytotoxicity (12–19).

## Expression of TNF and Its Receptors TNFR1 and TNFR2

TNF is mainly produced by immune cells, e.g., monocytes, macrophages, and T- and B-cells (20). Non-immune cells, such as keratinocytes, astrocytes, endothelial, and epithelial cells but also cancer cells can also express TNF (20). TNF production is highly inducible (up to 10.000 fold). Members of the NFAT-, NFκB-, and basic region-leucine zipper transcription factor families control TNF production on the transcriptional level and ERK1/2, p38MAPK and JNK signaling at the posttranscriptional level by modulation of mRNA stability and translation efficacy (20, 21). As TNF activates the MAP kinase signaling cascades and transcription factors of the NFκB family, TNF can induce its own transcription via both types of TNF receptors (16, 22–28). While TNFR1 is expressed in almost any cell type, TNFR2 expression is limited to myeloid cells, T- and B-cells and endothelial cells (29, 30). Although, sometimes several thousand molecules can be detected, TNFR1 expression levels are typically below 1,000 molecules per cell, especially in T cells (Table 1), limiting its detection with flow cytometry. Thus, lack of flow cytometric TNFR1 detection does not exclude functionally relevant TNFR1 molecule numbers. TNFR2 expression varies more and can reach ≥10^5^ molecules per cell in tumor cell lines (40).

**Table 1:** TNFR1 and TNFR2 expression in primary cells.

**Cell type**	**TNF binding sites per cell**			**References**
	**Total**	**TNFR1**	**TNFR2**	
Human umbilical cord vein (HUVEC) cells	1,500	n.d.	n.d.	(31)
Human SAC^a^-activated B-cells	6,000	n.d.	n.d.	(32)
Human neutrophils	3,000	n.d.	n.d.	(33)
Human neutrophils	6,000	n.d.	n.d	(34)
Peripheral T cells, healthy subjects	130/140	n.d.	n.d.	(35–37)
Peripheral T cells, MS patients	950/840	n.d.	n.d.	(35, 36)
Peripheral T cells, myasthenic patients	660	n.d.	n.d.	(37)
OKT3/IL2 activated T cells	600	n.d.	n.d.	(38)
PHA activated PBMCs	5,600	10–20%	80–90%	(39)

a *Staphylococcus aureus Cowan strain I*.

## TNF and Its Receptors in T Cell Biology

After its molecular cloning, TNFR2 was discovered to promote proliferation of thymocytes and peripheral T cells (41, 42). Subsequently, TNFR2 was recognized as a costimulator of naive CD8^+^ T cells *in vitro* and *in vivo* (43–46). Accordingly, TNFR2-mediated T cell costimulation is impaired in patients suffering from common variable immunodeficiency (47). At the molecular level, the costimulatory activity of TNFR2 has been associated with an increased expression of survival proteins such as survivin and Bcl-2 (44). However, the role of TNFR2 in CD8^+^ T cell regulation is presumably more complex, context-dependent, and goes beyond sole improvement of CD8^+^ viability. For example, in mice infected with respiratory influenza virus or acute lymphocytic choriomeningitis virus TNFR2 contributes to the contraction of the antigen-specific CD8^+^ T cell population (48, 49). In accordance with the counterintuitive proapoptotic TNFR2 activity in these models, TNFR2 deficient CD8^+^ T cells were less sensitive for TNFR1-dependent cell death and activation induced cell death *in vitro* (50, 51). As discussed above, TNFR2 can sensitize cells for TNFR1-induced cell death by depletion/degradation of protective TRAF2-cIAP/2 complexes but also activates the alternative and classical NFκB pathways, which upregulate antiapoptotic proteins and proliferation promoting factors. Thus, it is tempting to speculate that the balance of these two effects determines the outcome of TNFR2 activation in CD8^+^ T cells. Particularly, in situations where CD8^+^ T cells are protected TRAF2-cIAP1/2-independently from TNFR1-induced killing, the proliferation promoting effects of TNFR2 might dominate.

## The Relevance of TNF and Its Receptors for TREG Biology and TREG Function

Early on, it had been reported that administration of soluble TNF to neonatal non-obese diabetic (NOD) mice enhanced diabetes onset while reducing CD4^+^CD25^+^ T cell numbers in thymus and spleen. Treatment with anti-TNF antibodies resulted in opposite effects (52). Moreover, T cell transfer experiments of CD4^+^CD25^+^ T cells from TNF-treated neonatal mice displayed diminished inhibitory activity (52). Again in the NOD model, TNF inhibited Tregs via TNFR1 (53). Accordingly, TNF contained in the synovial fluids of rheumatoid arthritis (RA) patients was reported to impair Treg function by upregulation of protein phosphatase 1 and dephosphorylation of Foxp3 (54). Notably, the latter was restored in RA patients treated with the TNF neutralizing antibody Infliximab (54). Already earlier and in accordance with a Treg inhibitory effect of TNF, several reports showed a moderate but significant increase in Treg frequency in the peripheral blood of RA patients treated with the TNF neutralizing antibodies Adalimumab and Infliximab (55–57). Furthermore, exogenous soluble TNF inhibited the suppressive activity of Tregs derived from HBV patients (58). Likewise, TNF alone, or in combination with IL6, inhibited the suppressive activity of Tregs isolated from naïve mice *in vitro* (59).

However, by 2007 Chen et al. not only showed that TNFR2 is highly expressed on murine and human Tregs but also that TNFR2 supports Treg proliferation and maintenance of their suppressive activity (60–64). Indeed, TNFR2^+^ expression marks the most suppressive subset of Tregs (63). Consequently, various animal models, including models of inflammatory diseases and cancer, confirmed the relevance of TNFR2 for Treg proliferation and Treg activity (Table 2).

**Table 2:** *In vivo* evidence for TNFR2-dependent Treg functions.

**Model**	**Method**	**Effect**	**References**
TNFR2 KO mice	Cecal ligation and puncture	Reduced Treg expansion	(60)
TNFR2 KO nTregs	T cell transfer induced colitis	Loss of suppressive Treg activity	(65)
TNFR2 KO	EAE	Reduced Treg numbers and exacerbated disease	(66)
TNFR2 KO Tregs of EAE mice	Transfer in EAE mice	Loss of EAE inhibitory activity	(66)
TNFR2 KO bone marrow reconstitution	B16F10 metastasis	Reduced tumor Tregs and metastasis	(67)
TNFR2 KO mice	Friend virus-induced Vß5^+^ Treg expansion	Loss Vß5^+^ Treg expansion	(68)
Wt mice	TNFR2 agonist	Vß5^+^ Treg expansion	(68)
Wt mice	TNFR2 agonist	Treg expansion	(69)
Wt mice	TNFR2 agonist priming	Treg expansion and protection from GvHD	(69)
TNFR2 KO Tregs	Treg transfer-mediated GvHD protection	Loss of GvHD protection	(70)
Wt Tregs	TNFR2 blocking antibody in Treg transfer-mediated GvHD protection	Loss of GvHD protection	(70)
Wt mice	TNFR2 agonist in collagen induced arthritis	Increased Treg number and reduced disease score	(71)
Wt and TNFR2 KO mice	TNFR2 agonist	Treg expansion in naïve mice	(72)

Noteworthy, adoptive transfer experiments with antigen-specific Teffs and Tregs revealed that effector T cells promote the expansion of sub-optimally proliferating antigen-stimulated Tregs in a TNF-dependent manner (62, 73). Similarly, Vβ5^+^ Tregs, recognizing mouse mammary tumor virus encoded superantigen, expand after Friend virus infection due to TNFR2 activation by CD8^+^ expressed membrane TNF (68). Thus, the capability of T cell expressed TNF to promote Treg proliferation and activation via TNFR2 may represent a negative feedback mechanism to terminate inflammation.

**Figure 2:**
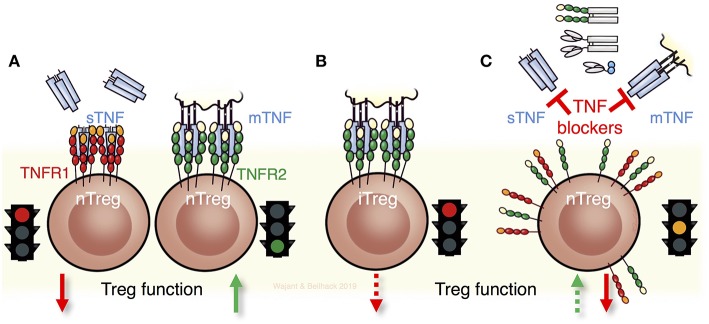
TNF and its receptors for Treg biology and Treg function. **(A)** Soluble TNF (sTNF) can impair the maintenance and function of thymic derived naturally occurring Tregs (nTregs) via TNFR1. In contrast, stimulation of TNFR2 expands and fosters the function of nTregs. **(B)** Notably nTregs and induced Tregs (iTregs) respond differently to TNF. Triggering of TNFR2 in iTregs diminishes their stability and function. **(C)** The seemingly contradictory results obtained with anti-TNF biologicals that are in current clinical use such as antibodies, antibody-fusion proteins, or Fab' fragments can be ascribed to the different effects of TNF on the two receptors TNFR1 and TNFR2. Consequently, neutralizing TNF and not directly targeting its receptors can result in complex scenarios by exerting detrimental and beneficial effects on Tregs, dependent on which receptor is being engaged and whether nTregs or iTregs, or both, are implicated.

The seemingly conflicting data on the proliferation and activity of Tregs via targeting TNF or TNFR2 might be related to two obvious scenarios:

First, neutralization of TNF might inhibit without discrimination detrimental and beneficial effects of TNF on Tregs that separates with the two TNF receptors (Figure 2). Evidence supports TNFR1 mediated negative effects on Tregs. TNFR1 deficiency increased Treg activity (53) and Tregs deficient for FLIP, a major inhibitor of TNF-induced apoptosis, have extremely low Treg numbers and develop a scurfy-like phenotype (74). Notably, TNFR2 markedly improves myeloid derived suppressor cell survival via FLIP upregulation (75). Opposing effects of the two TNF receptors have also been reported regarding the suppressive effect of Tregs on effector T cell proliferation *in vitro* (76). While TNFR1 deficiency in Tregs resulted in enhanced suppressive activity, TNFR2 deficient Tregs almost completely lost their suppressive potential.

Another factor contributing to the seemingly inconsistency in the available literature on the role of TNF in Treg biology is that nTregs and iTregs respond differently to TNF (Figure 2). Indeed, TNF neutralization in an EAE model increased Treg levels due to the reversal of an inhibitory effect of TNF on TGFß-induced iTreg differentiation (77), while nTregs remained unaffected. Noteworthy, TNF inhibited iTreg differentiation also via TNFR2 (77). Accordingly, restoration of Treg function in RA patients treated with Infliximab has been traced back to an emerging and unusual CD62L^−^ Treg population that after TNF blockade differentiates via TGFβ from CD4^+^CD25^−^ cells of RA patients but not of healthy individuals (78).

## TNFR2 Signaling in Regulatory T Cells

**Figure 3:**
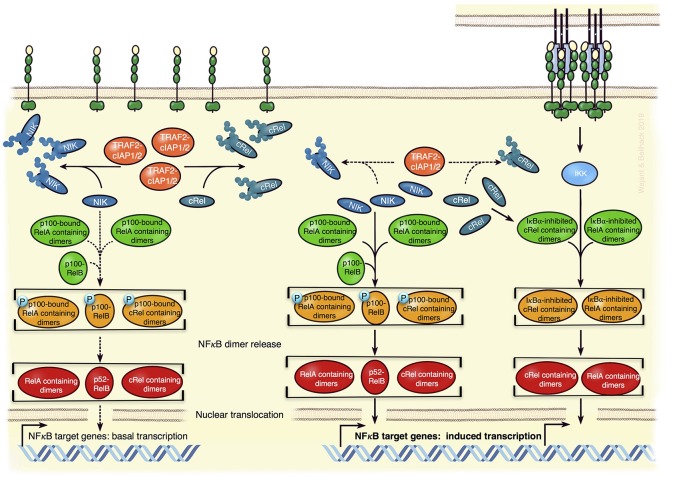
Model of TNFR2-mediated regulation of NFκB transcriptions factors in Tregs. In absence of appropriate exogenous stimuli, the classical NFκB pathway is not active (**Left**). Constitutively active cytosolic TRAF2-cIAP1/2 complexes K48-ubiquitinate NIK and cRel triggering so the proteasomal degradation of these proteins (**Left**). This not only dampens the activity of the NIK-dependent alternative NFκB pathway to low basal levels but also diminishes the amount of cRel-containing dimers, which can be activated via the classical NFκB pathway. Since p100 can inhibit cRel- and RelA-containing NFκB dimers, it might also reduce the responsibility of the classical NFκB pathway. TNFR2 activation by memTNF results in the recruitment of TRAF-cIAP1/2 complexes and activation of the IKK-dependent classical NFκB pathway (**Right**). The depletion of the cytosolic TRAF2-cIAP1/2 complexes associated herewith leads to reduced degradation of NIK and cRel, thus (i) to enhanced alternative NFκB signaling and (ii) more cRel-containing NFκB dimers that can respond to the classical NFκB pathway (**Right**). The model is based on what is known about the specific functions of NFκBs in Tregs and the mechanisms of TNFR2 signaling in general.

Already in 2002, high TNFR2 expression was reported on human CD4^+^CD25^+^ thymocytes, which showed T cell suppressive activity after polyclonal expansion (79). The first reports demonstrating the importance of TNFR2 for Treg functions, however, were published only 5–6 years later (60, 61, 80). Although regulation of Tregs has meanwhile become the most intensively studied *in vivo* activity of TNFR2, limited knowledge exists about the molecular mode of action in Tregs. Based on what is known about TNFR2 signaling in other cell types, without claim of completeness, three possible mechanisms appear plausible:

First, TNFR2-induced activation of NFκB transcription factors (Figure 3). Activation of the classical and alternative NFκB pathway by TNFR2 has been demonstrated in a variety of cell types and these pathways are also stimulated by the TNFR2-related TNFRSF receptors CD27, OX40, and GITR, all been implicated in Treg development or survival. Therefore, it is tempting to speculate that TNFR2-induced NFκB signaling is also involved in the control of Treg expansion/activity. In fact, the NFκB subunit cRel, with its well-established role in thymic Treg development and which has also been implicated in iTreg generation (81) has just recently been identified, along with p65 (RelA), as a crucial factor for the maintenance and functionality of mature nTregs and iTregs (82, 83). Activation of cRel- and p65-containing NFκB dimers is typically triggered by the classical NFκB pathway. Despite normal or slightly increased Treg numbers in spleen and lymph nodes, mice with cRel or p65 deficient Foxp3^+^ Tregs showed mild (cRel deficient Tregs) or significant but slowly progressing (p65 deficient Tregs) lymphoproliferative disease (83). This points to a role of cRel and p65 for the suppressive activity of Tregs. Indeed, in contrast to wild type Tregs, Tregs lacking cRel or p65 were unable to rescue mice from T cell transfer-induced colitis (83). Mice double deficient for p65 and cRel in Tregs succumbed early to a scurfy-like (Foxp3 defective) phenotype (83). Although, cRel deficiency did not impair iTreg formation, cRel is also here relevant, because absence of cRel and p65 in CD4^+^ T cells impaired iTreg induction (83, 84). Tregs can also be categorized in two distinct functional subsets, resting Tregs (rTregs) in lymphoid tissue, and activated Tregs (aTreg) with reduced Foxo1 expression, migrating to inflamed tissues including cancer (85). Now, cRel but not p65 turned out as important for aTreg differentiation and tumor development (82). Inducible p100 processing, which results in the conversion of p100-RelB complexes to a p52-RelB NFκB dimers (Figure 3), is the central step in the alternative NFκB pathway. Mice with p100 deficient Tregs also develop a mild autoimmune syndrome, which depends on RelB and correlated with increased Treg numbers with reduced suppressive activity (86). Notably, interaction with the ankyrin domain of p100 can also inhibit cRel- and p65-containing NFκB dimers (87), whereby p100 binds cRel-containing dimers more preferential than RelA-containing dimers (88). Thus, TNFR2 via activation of the alternative NFκB pathway has the potential to crosstalk into the classical NFκB pathway. Considering that TNFR2 seems to be more important than TNFR1 in Tregs and because TNFR2, in contrast to TNFR1, triggers not only the classical NFκB pathway but also the alternative NFκB pathway, the following scenario appears plausible: TNFR2 (or other p100 processing-triggering TNFRSF receptors) triggers/maintains Foxp3 expression and Treg suppressive activity by stimulating both NFκB pathways yielding in the coordinated activation of cRel, RelB, and RelA-containing NFκB dimers (Figure 3).

A second possible mode of TNFR2 signaling in Tregs is based on the ability of TNFR2 to sequester and degrade TRAF2. Depletion of cytoplasmic TRAF2-cIAP1 and TRAF2-cIAP2 pools may not only result in the accumulation of NIK and activation of the alternative NFκB pathway but might also promote other signaling events, which are inhibited in unstimulated cells by TRAF2-mediated degradation. In fact, TRAF2 and cIAPs antagonize proinflammatory signaling in myeloid cells by promoting proteasomal degradation of cRel and IRF5 (89). Therefore, TNFR2-induced depletion of cytosolic TRAF2-cIAP1/2 complexes has the potential to increase cRel levels (Figure 3).

Thirdly, it has been suggested that TNFR2 elicits its effect on Tregs not directly by triggering intracellular signaling pathways but indirectly after shedding from the plasma membrane and inhibiting soluble TNF (80). A functional relevant robust TNF neutralizing effect of the soluble TNFR2 ectodomain, however, is hard to reconcile with the very low affinity of monomeric TNFR2 for TNF (90).

## Preclinical and Clinical Evidence for the Usefulness of Therapeutic TREG Targeting via TNFR2

Adoptive transfer of Tregs is a straightforward strategy to exploit the overwhelming immunotherapeutic potential of this cell type, which is being explored in clinical studies (91). Purification/enrichment and *ex vivo* expansion of stable and functional Tregs are crucial factors complicating the applicability and success of therapeutic adoptive Treg transfer (92). In accordance with the crucial role of TNF-TNFR2 signaling in Treg biology, two recent studies demonstrated beneficial effects of targeting of the TNF-TNF receptor system in *ex vivo* Treg expansion protocols. Using a not further specified agonistic TNFR2 antibody, Okubo et al. demonstrated that the additional activation of TNFR2 in standard Treg expansion protocols conferred improved suppressive activity while reducing Treg heterogeneity (93). Furthermore, using the TNFR2-specific mAb MR2-1 as an agonist, TNFR2 signaling promoted the expansion of low purity MACS-isolated Treg preparations to stable homogenous Treg populations (94). Therefore, TNFR2 agonists may potentially improve *ex vivo* Treg expansion methods for clinical applications.

**Figure 4:**
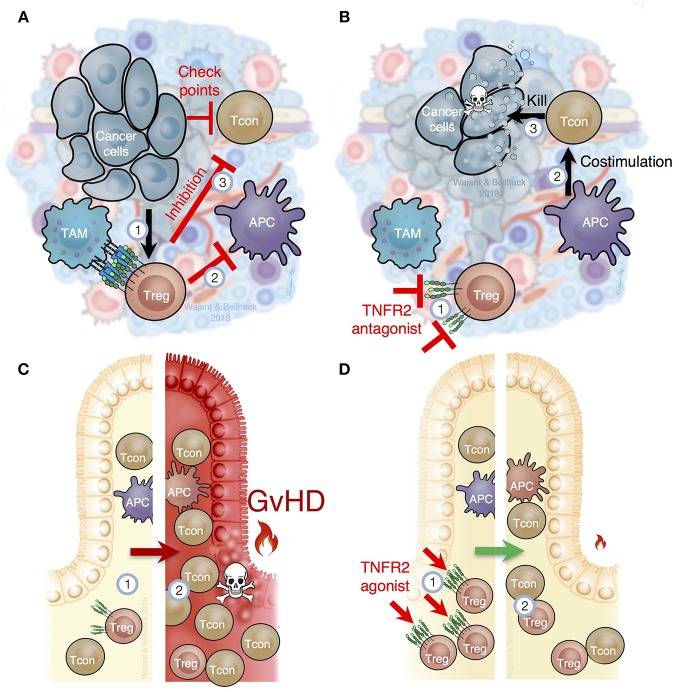
TNFR2 is critical for Treg function in cancer and inflammation. **(A)** In the tumor microenvironment, immune cells, such as tumor associated macrophages (TAM), stroma, and tumor cells, produce TNF, which (1) attracts and stimulates Tregs and myeloid derived suppressor cells (MDSC). TNFR2-mediated Treg activation prevents optimal costimulation by antigen presenting cells to trigger cytotoxic tumor-specific T cell responses (2) and, importantly, prevents T cell mediated tumor lysis through several immune checkpoints (3). **(B)** Selective inhibition of TNFR2 or depletion of TNFR2^+^ Tregs in tumors (1) would improve costimulation by tumor antigen presenting cells (APC) to activate cancer specific immune responses (2), abolish the blockade of cancer specific immune responses within the tumor tissue (3) and, thus, reactivate cytotoxic T cells to destroy cancer cells. **(C)** In recipients of allogeneic hematopoietic cell transplantation (HCT), the underlying disease, intensive therapy and host conditioning systemically reduce Tregs (1). After allogeneic HCT Tregs are overwhelmed to control alloreactive T cells (2), which cause acute graft-vs.-host disease (GvHD). **(D)** TNFR2-specific agonists stimulate Tregs in secondary lymphoid organs and peripheral tissues (1). Increased Treg numbers and function support tissue homeostasis and can contain excessive T cell responses (2) as they occur in acute GvHD or other inflammatory diseases.

First decisive evidence for the *in vivo* drugability of the TNF-TNFR2 interaction in nTregs stems from mouse experiments of GvHD and collagen-induced arthritis (CIA). Employing a TNFR2-selective nonameric variant of murine TNF, several groups found that exogenous TNFR2 stimulation suffices to expand Treg numbers in mice (68, 69, 71, 72). TNFR2 agonist-induced Treg expansion protected mice from subsequent allogeneic hematopoietic cell transplantation (HCT)-induced GvHD, while preserving graft-vs.-leukemia activity (Figure 4) (69). Inhibiting the TNF-TNFR2 interaction blocked Treg activity in GvHD (70). TNFR2-promoted Treg expansion also attenuated the clinical score of mice suffering from CIA (71). In accordance with these findings, Pierini et al. reported that *in vitro* TNF priming in the presence of IL2 enhances TNFR2-dependent murine Treg activation and proliferation resulting in Tregs providing superior protection from GvHD (95).

The microenvironment of most tumors is highly enriched with TNF producing cells such as macrophages, T cells, and fibroblasts and often contains increased Treg numbers that crucially contribute to tumor immune escape and tumor progression (Figure 4). Based on the compelling evidence that TNF-TNFR2 interaction stimulates Treg activity, various studies addressed the feasibility of blocking TNFR2 therapeutically in animal cancer models. In one study, loss of tumor immunity against secondary tumors was traced back to CD103^+^ effector Tregs with high TNFR2 expression (96). *In vitro*, TNF induced TNFR2-mediated effector Treg expansion and their transfer suppressed antitumoral CD8^+^ T cell responses (96). Likewise, increased effector Treg frequencies were found in peripheral blood samples of colon rectal carcinoma and hepatocellular carcinoma patients. Again, these Tregs were significantly enriched *in vitro* in response to TNF (96). Moreover, soluble hTNFR2-Fc enhanced the antitumor activity of cyclophosphamide and further reduced effector Treg numbers in mice bearing CT26 tumors without affecting CD8^+^ T cell activation (96). Also in CT26 bearing mice, blockade of TNFR2 signaling with the antagonistic antibody M861 reduced TNFR2^+^ Treg frequency within the tumor microenvironment and enhanced the immune stimulatory activity of CpG oligodesoxynucleotides (97). Notably, the antagonistic TNFR2 antibody TR75-54.7 inhibited growth of 4T1 tumors more efficiently than the antagonistic CD25 mAb PC61 (97). Using a novel antagonistic TNFR2 antibody, Torrey et al. demonstrated that TNFR2 blockade in ascites of ovarian cancer patients result in reduced Treg numbers and increased effector T cell frequency (98).

Patients suffering from acute myeloid leukemia (AML) display increased Treg numbers in the peripheral blood and bone marrow (99), which correlate with poor prognosis (100). The majority of these Tregs strongly express TNFR2 and efficiently migrate into the bone marrow (101). In AML patients subjected to epigenetic therapy, a reduction of TNFR2^+^ Tregs have been observed in the bone marrow of responders compared to non-responders whereas there was no difference in TNFR2^−^ Tregs before and after treatment (101).

## Preclinical Development of TNFR2-Targeting Reagents

The goal of TNFR2 targeting clearly depends on the considered disorder. While inhibiting TNFR2 activities in Tregs or even destroying Tregs may be the goal in cancer, stimulating TNFR2 may be the aim to treat inflammatory conditions or inflammation-associated cancer to improve immune suppression by Tregs.

## Preclinical Drugs With TNFR2-Inhibitory Activity

**Figure 5:**
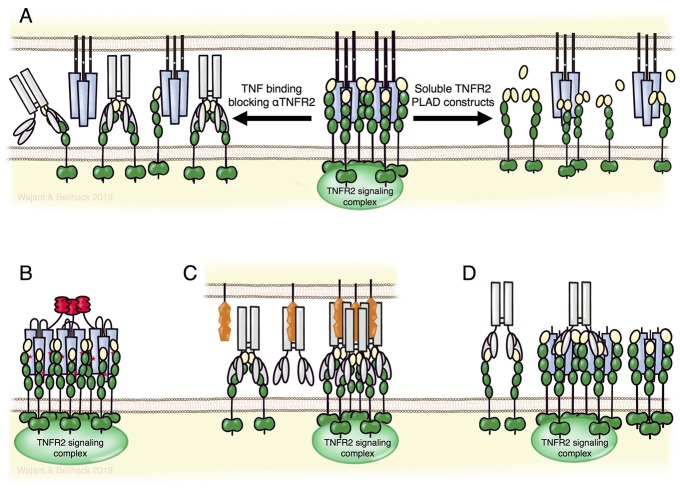
TNFR2 targeting biologicals. **(A)** TNFR2-specific antibodies blocking TNF binding (left part) or fusion proteins of the TNFR2 PLAD domain (right part) can block the formation of TNFR2 signaling complexes without directly affecting TNFR1-related activities. Lower panel: nonameric variants of TNFR2-specific TNF mutants **(B)** and FcγR-bound agonistic anti-TNFR2 antibodies **(C)** allow specific activation of TNFR2. *Per se* non-competitive non-agonistic TNFR2-specific antibodies that oligomerize poorly active soluble TNF-TNFR2 complexes can potentiate TNFR2 signaling **(D)**. For details see text.

In principle, TNFR2 activation might be prevented pharmacological by use of one of the various approved TNF-neutralizing biologicals for the treatment of autoinflammatory diseases. However, this would also inhibit TNFR1 signaling counteracting the beneficial effects of reduced TNFR2 activity. In the immunotherapy of cancer, for example, inhibition of TNFR2 might help to break tumor-associated immune tolerance by reducing Treg activity. The intended stimulation of anti-tumor immunity, however, would suffer from inhibiting proinflammatory TNFR1 activities, too. Selective inhibition of TNFR2 is obviously possible with TNFR2-specific antibodies blocking TNF binding and lacking intrinsic TNFR2-stimulating activity (Figure 5, upper panel). The development of antagonistic anti-TNFR2 antibodies appears, at first glance, simple. Indeed, various reports described the use of antagonistic TNFR2-specific antibodies *in vitro* on non-hematopoietic cells. *In vivo*, however, the situation might be complicated by the presence of immune cells and immune cell-associated expression of Fcγ-receptors (FcγRs). Various preclinical *in vivo* studies demonstrated that FcγR-binding can act as an all-dominant factor that determines the agonistic activity of TNFRSF receptor-specific antibodies and even converts antagonistic antibodies into strong TNFRSF receptor agonists (3). The FcγR binding-dependent agonistic activity of TNFRSF receptor-targeting antibodies presumably reflects the fact that membrane-anchoring of antibodies promotes the secondary oligomerization of initially formed antibody-TNFRSF receptor complexes, which is needed for full receptor activation (3). In any case, this issue should be evaluated in course of the development of antagonistic anti-TNFR2 antibodies and could necessitate the use of antibody isotypes/variants devoid of FcγR binding.

Inhibition of TNFR2 activity might also be achieved by non-antibody based drugs. For example, Tang et al. identified progranulin as a high-affinity competitor of TNF binding to TNFR2 (102). However, progranulin also competes with TNF for TNFR1 binding and with TL1A for DR3 binding (102, 103). Thus, progranulin based TNFR2 blockers let expect similar limitations as discussed above for TNF-neutralizing reagents. Moreover, several independent groups failed to observe inhibition of TNF-TNFR1/2 interaction by progranulin (104–106). In fact, it has been reported that progranulin rather enhances, than blocks, TNF-induced TNFR2-mediated proliferation of Tregs (107). In the absence of ligand, TNFR1, TNFR2 and several other receptors of the TNFRSF undergo homotypic interaction without activating cellular signaling pathways (108–110). This is mediated via the N-terminal pre-ligand assembly domain (PLAD) and is required for efficient ligand binding. Accordingly, dimeric Fc and GST fusion proteins of the TNFR1 PLAD inhibit TNFR1-promoted pathologies in preclinical models (110–113). Because of the homotypic PLAD-PLAD interaction, fusion proteins of the TNFR2-PLAD may act as TNFR2-specific antagonists leaving TNFR1 signaling intact (Figure 5, upper panel).

## Preclinical Drugs for Selective TNFR2-Stimulation

Activation of TNFR2 can be achieved by recombinant variants of its natural ligands TNF and LTα or by agonistic antibodies or antibody mimetics. Two aspects require consideration for recombinant ligand variants for TNFR2 stimulation: first, receptor selectivity, as TNF (and LTα) interacts with TNFR2 and TNFR1; second, limited ability of soluble TNF trimers to stimulate TNFR2 signaling. The challenge of TNFR2 selectivity had been solved early, by various groups identifying mutations conferring selectivity for one of each of the two TNF receptors. Furthermore, oligomerization of soluble TNF trimers potentiates their ability to stimulate TNFR2 (5). This knowledge triggered our rational design of human and mouse TNF fusion proteins that comprise three ligand trimers and act as very potent TNFR2 agonists. To obtain three covalently linked TNF trimers the small trimerization domain of tenascin-C was genetically linked to a triplet of TNFR2-specific TNF protomers separated by peptide linkers long enough to allow intramolecular self-assembly (5, 69). *In vitro* binding and functional studies proved high selectivity of these agonistic fusion proteins for TNFR2 and, accordingly, in no toxicity in mice treated repeatedly with high doses of the murine TNFR2-specific variant (69).

Besides recombinant soluble TNFSF ligand variants, agonistic antibodies are the classical approach to activate receptors of the TNFRSF (Figure 5, lower panel). Based on superior pharmacokinetics and the broad experience in antibody production and development, agonistic antibodies remain the first choice to accomplish therapeutic activation of TNFRSF receptors. Indeed, various agonistic antibodies targeting immune stimulatory or cell death-inducing members of the TNFRSF are currently under investigation in clinical trials for cancer therapy. As discussed above for the development of antagonistic TNFR2-specific antibodies, one has to consider again the possibility of antibody binding to FcγRs and the possible agonism-boosting and immune cell-stimulating effects of these interactions. Since agonistic TNFRSF receptor-specific IgG antibodies frequently achieve only maximum activity upon FcγR-binding, such antibodies risk to trigger destruction of targeted cells instead of receptor activation. Thus, TNFR2 targeting with such antibodies *in vivo* could rather deplete Tregs instead of promoting Treg expansion. Therefore, antibodies with a high intrinsic, FcγR binding-independent agonistic activity or Fc domain-mutated antibodies preferentially binding to inhibitory FcγRs may account for the best strategy to achieve TNFR2 activation with agonistic antibodies *in vivo*.

An interesting option to achieve TNFR2 activation *in vivo* is the use of non-competitive antibodies modifying the receptor response to soluble ligand trimers. It has been found that some non-competitive and *per se* non-agonistic antibodies against TNFRSF receptors can strongly enhance receptor activation upon soluble ligand binding presumably via aggregation of otherwise poorly active trimeric ligand-receptor complexes (Figure 5, lower panel) (114, 115). This mode of action has also been demonstrated for the TNFR2-specific mAb 80M2 (4). Clinical development of a TNFR2-specific antibody of this type may have two advantages: first, the “agonistic” activity would be fully independent from FcγR-binding and second, the “agonistic” activity would be closely spatiotemporally linked to sites where TNF is actively expressed.

## Targeting TNFR2 to Enhance Treg Function in GvHD

The pathophysiologic sequelae of acute GvHD follows a spatiotemporally orchestrated pattern of disease initiation and an ensuing effector phase (116–120). TNF plays a crucial role in all these events through several mechanisms. Host conditioning triggers an instant TNF release by host macrophages (121) which might enhance maturation of host type antigen presenting cells (APCs), the expression of MHC molecules (122) and T cell adhesion to APCs (123). TNF furthermore provides costimulatory signals to naïve CD4^+^ T cells and CD8^+^ cytotoxic T lymphocytes (44, 124–126). TNF, together with IL1β, also enhances TNF expression by freshly activated alloreactive T cells constituting a feed-forward-loop of TNF release (127–129). However, only in recent years it has become clear that TNF also triggers anti-inflammatory feedback loops, e.g., by stimulation of Tregs and myeloid derived-suppressor cells via TNFR2 (see previous paragraphs). After initial priming in secondary lymphoid tissues, alloreactive effector T cells home into GvHD target tissues (116, 117, 130, 131). Upon allorecognition, tissue infiltrating donor T cells release TNF, which can cause epithelial damage (120, 132). Fn14, a tissue damage-induced receptor of the TNFRSF, sensitizes intestinal epithelial cells and renders them particular susceptible to TNF-dependent apoptosis (133). This may also explain, at least in part, why the intestinal tract is a primary target for GvHD tissue damage. The consequent disruption of the barrier function of the gut epithelium results in a vicious cycle of exacerbating GvHD (134).

In patients, systemic TNF release of >100 pg/mL in the first 3 months after allo-HCT strongly correlated with acute GvHD, veno-occlusive disease, endothelial leakage syndrome, and interstitial pneumonitis (128, 129). Also, it was found that levels of shed TNFR1 and TNFR2 correlate with systemic TNF concentrations and allo-HCT related complications (135, 136). Subsequently, it has been furthermore found that a strong increase of soluble TNFR1 (sTNFR1) 7 days after allo-HCT correlated with GvHD incidence and severity and patient survival (137, 138). These results lead to the integration of sTNFR1, together with interleukin-2-receptor-alpha, interleukin-8, and hepatocyte growth factor, into a proposed serum biomarker panel for GvHD diagnosis and prediction of survival (139).

The detrimental effects of TNF on GvHD pathogenesis provided a clear rationale to test TNF-inhibitors in allo-HCT. Indeed, TNF blockade prevented acute GvHD in most mouse models but may also affect graft-vs.-leukemia activity as transplantation of TNFR1 deficient donor CD8 T cells resulted in an increased leukemia relapse after allo-HCT (120, 121, 127, 140). Based on these data, several clinical studies were initiated to test TNF inhibitors for the treatment of acute GvHD or as a preemptive therapeutic approach to prevent the onset of acute GvHD. Importantly, the TNF blocking antibody infliximab failed in clinical trials, both in a treatment setting and in a preemptive therapy approach, and might even increase bacterial and fungal infections (141, 142). Although etanercept, a Fc fusion protein of the TNFR2 ectodomain, in combination with high-dose steroids showed initially promising response rates in GvHD patients, it neither improved survival in comparison to control subjects nor showed it a beneficial activity in a prophylactic setting (143–145).

Blocking TNF does not only inhibit the primarily TNFR1-mediated proinflammatory TNF activities but also the predominantly TNFR2-mediated protective effects. The ambivalence of therapeutically targeting TNF is emphasized by the experience with TNF inhibitors in the treatment of autoimmune diseases. Clearly, TNF blockers have been a game-changer for the treatment of inflammatory diseases such as rheumatoid arthritis and colitis showing high response rates in many patients making them the commercially most successful biologicals on the market. However, many patients do not respond to TNF inhibitors and TNF blockers may even exacerbate inflammation in other diseases, e.g., heart failure or multiple sclerosis (146, 147). This emphasizes that, despite the prominent perception of TNF as a potent proinflammatory cytokine, TNF can exert important immunosuppressive functions, likely depending on the underlying disease and the involved immune regulatory cells.

As pointed out above, an important mechanism explaining these opposing outcomes of TNF-inhibition is the dichotomy of TNFR1- and TNFR2-mediated effects. Therefore, directly addressing TNFR1 or TNFR2 as therapeutic targets through TNFR1 antagonists or TNFR2 agonists appears as an attractive strategy to improve current clinical practice of GvHD treatment. So far, this strategy has been tested in preclinical mouse models employing TNFR2-selective agonists. TNFR2-mediated *in vivo* expansion of Tregs could prevent acute GvHD (69). Notably, fostering Treg numbers and their function may not only counterbalance excessive inflammation but may also improve tissue regeneration (148, 149). Restoration of tissue homeostasis in GvHD target tissue may prove as a key mechanism to improve outcomes in patients undergoing allo-HCT.

Conclusively, therapeutically targeting of TNFR2 in patients appears as a highly promising approach to either propagate donor Tregs *in vitro* or, importantly, to enhance Treg activity by expanding TNFR2^+^ Tregs in patients before allo-HCT to prevent GvHD. This attractive approach promises to reduce the risk for GvHD while allowing for alloimmune responses against remaining leukemia cells or to allow for efficient immune control of opportunistic infections. More caution will be warranted to employ TNFR2-agonists at the time of donor lymphocyte infusion or at the onset of GvHD. Clearly, the stimulatory effects of TNFR2 on Tcons require careful assessment in preclinical *in vivo* models before TNFR2 agonists will enter clinical trials.

## Author Contributions

All authors listed have made a substantial, direct and intellectual contribution to the work, and approved it for publication.

### Conflict of Interest Statement

Through the department of technology transfer of Würzburg University, HW and AB have filed patent applications regarding the development and generation of TNFR superfamily-addressing reagents.
